# Working to enhance the accessibility of Disease Models & Mechanisms

**DOI:** 10.1242/dmm.049470

**Published:** 2022-01-31

**Authors:** Rachel Hackett, E. Elizabeth Patton

**Affiliations:** 1 The Company of Biologists, Bidder Building, Station Road, Histon, Cambridge CB24 9LF, UK; 2 MRC Human Genetics Unit and Cancer Research UK Edinburgh Centre, Institute of Genetics and Cancer, The University of Edinburgh, Western General Hospital, Crewe Road South, Edinburgh EH4 2XU, UK

## Abstract

**Summary:** The DMM Editor-in-Chief discusses the importance of accessibility to published research and outlines the implications of new Read & Publish agreements for the DMM community.

In February 2022, the Budapest Open Access Initiative (BOAI) will celebrate its 20th anniversary. This important initiative offered the first definition of Open Access (OA) – creating “a statement of principle, a statement of strategy, and a statement of commitment” (https://www.budapestopenaccessinitiative.org/read/). The overall purpose of the initiative was to accelerate progress in international efforts to make Research articles immediately and freely available to all.

**Glossary**

**Altmetrics:** Altmetrics track the attention that Research articles receive online, including data from social media such as Twitter and Facebook, traditional media, blogs, and online reference managers such as Mendeley and CiteULike (https://www.altmetric.com/about-altmetrics/what-are-altmetrics/).

**Article processing charge (APC):** to provide Open Access (OA), an APC fee is charged to the authors or funders of the research.

**Directory of Open Access Journals (DOAJ):** the DOAJ is an online directory that indexes high-quality, OA, peer-reviewed journals. The DOAJ Seal is awarded to journals that demonstrate best practice in OA publishing. Around 10% of journals indexed in DOAJ, including DMM, have been awarded the Seal.

**Gold OA:** different OA types can be described using a colour system. In the Gold OA model, the publisher makes all articles available for free immediately on the journal's website, usually under some form of Creative Commons license.

**Open Access (OA):** “A set of principles and a range of practices through which research output is distributed online, free of cost or other access barriers.”

**Plan S:** a funder initiative. Launched in 2018, the plan is supported by cOAlition S, an international consortium of research funders. Plan S requires that, from 2021, scientific publications that result from research funded by public grants must be published in compliant OA journals or platforms.

**Read & Publish agreements:** these agreements, between publishers and libraries/consortia, generally offer unlimited ‘read’ access to subscription journals plus uncapped fee-free OA publishing of Research articles.

Since this bold step, the scholarly communication ecosystem has changed profoundly over the subsequent two decades, with an abundance of new publishing and subscription models from commercial and not-for-profit publishers. The increasing importance of OA has presented challenges and opportunities for libraries, funding organisations, publishers and researchers alike. One thing is certain, however – OA will continue to play an important role in the future of publishing.

The Company of Biologists is a not-for-profit publishing organisation dedicated to supporting and inspiring the biological community worldwide. The Company has been committed to OA publishing since 2004, believing that it benefits science through wider and faster dissemination, increased readership and an acceleration in the sharing of quality research. We also believe that OA publishing is fairer to the funders – often including the taxpayer and charities.

The Company launched Disease Models & Mechanisms (DMM) in 2008 as a subscription journal, electing to make the transition to a fully OA journal in 2011. The Directory of Open Access Journals (DOAJ) now lists more than 17,000 OA peer-reviewed journals, including DMM, which has the DOAJ Seal. DMM is a Gold OA journal, with costs covered by the authors or their funders paying an article processing charge (APC).

For DMM, accessibility is one of the twin pillars underpinning its ethos (Patton, 2021). We believe that the results of scientific research should be accessible to all. It is crucial that – whatever their funding body or financial status – DMM authors can ensure that their research is freely accessible to all, including other researchers, clinicians, patients and their families and advocates, their funders and the wider public.

For an increasing number of researchers, OA is very important or even essential to ensure that their research is read by their peers and has maximum impact in their field. The number of funders mandating OA publication will only continue to grow over the coming years. For example, cOAlition S, a coalition of 20+ (largely European) funders, implemented new OA mandates from January 2021 under an initiative called Plan S (https://www.coalition-s.org/). Plan S offers a number of routes by which a journal can be compliant with its policies, including full (Gold) OA and transformative arrangements.

Under Plan S, full (Gold) OA journals such as DMM are required to offer APC waivers for authors from low-income economies and discounts for authors from lower-middle-income economies, as well as waivers and discounts for other authors with demonstrable needs. DMM has long held this policy, and indeed has a more generous policy in that authors from lower-middle-income economies are entitled to full waivers.

A journal must also provide transparent metrics on both its pricing model and its editorial process. DMM already provides article-specific metrics on usage, citations and online attention (as measured by Altmetrics) for published articles. DMM now also provides other transparent metrics about what authors should expect when they submit to our journal, such as decision times, acceptance rates and speed to publication: these can be found at https://journals.biologists.com/dmm/pages/transparent-metrics. Some of these metrics are required by Plan S, but we will, in time, add additional metrics that we think will be of interest to our community.

As well as our waiver option, since 2021, authors based at the University of California and Max Planck Society Institutes have been entitled to discounted or fee-free OA publication, respectively, in DMM, as part of Read & Publish agreements offered by The Company of Biologists (https://www.biologists.com/library-hub/read-publish/). These cost-neutral Read & Publish agreements also give institutions the usual subscription access for their readers to all subscription content from our sister journals Development, Journal of Cell Science and Journal of Experimental Biology.

As of 2022, DMM is now included in a growing number of additional Read & Publish deals, meaning that more researchers are eligible to publish their OA articles in DMM for free. You can find out whether your institute is included at https://www.biologists.com/library-hub/read-publish/participating-institutions/ (note that DMM is included in the five-journal packages). And we hope to see many more institutes signing up to our Read & Publish deals in the future (see how our Read & Publish agreements benefit researchers in the infographic). We believe that this will ensure that the transition towards OA is smooth, transparent, affordable and sustainable for all.

**Figure:**
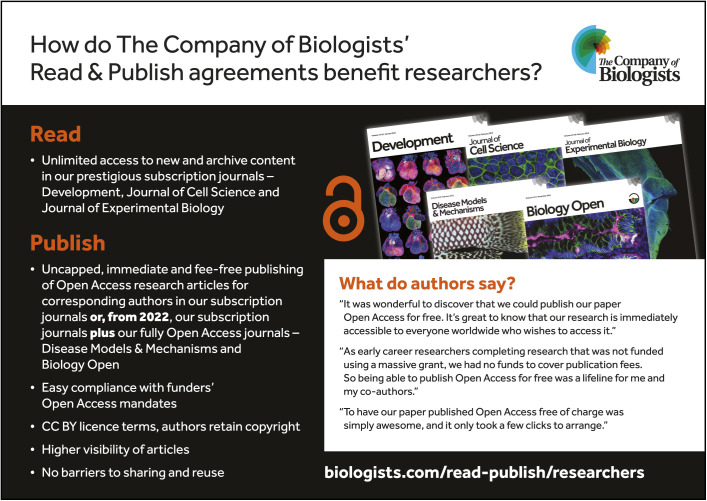

At DMM, we have always put the interests of our community at the heart of our publishing decisions – in terms of both providing valuable services to authors and ensuring that the income generated benefits the community through our charitable activities (for more details, see Box 1; see Box 2 for useful related links). Whatever the future of publishing may hold, you can rest assured that The Company of Biologists and DMM will remain committed to supporting its authors, reviewers and readers. DMM would also like to take the opportunity, as we do at the start of every year, to thank our reviewers for their time, expertise and dedication. The names of all our 2021 reviewers, including their co-reviewers, are listed in the supplementary information.

**Box 1. Added value: supporting biologists, inspiring biology**

One of the principles of Plan S is that any Open Access fees charged ‘must be commensurate with the publication services delivered’. What does this mean in the context of The Company of Biologists’ journals? By far the biggest costs for the journal are in our people: our academic editors and in-house teams of administrators, editors and production staff. Between them, they manage our editorial and peer-review workflows, provide support to authors and reviewers, commission and edit news/review-type articles, copy edit and proofread accepted papers (and process their associated figures, movies and supplementary data), and check every accepted manuscript for both plagiarism and image manipulation. All these processes – alongside the platforms, partners and technologies needed to support them – help to ensure that the papers we publish conform to the high standards you should expect from a journal like DMM.

The Company of Biologists is a not-for-profit publisher and a registered UK charity, so while our revenues do currently exceed our publishing costs, those profits are used to support the biological community worldwide. Each year, we provide hundreds of meeting grants and travelling fellowships to conference organisers and early-career researchers, respectively, as well as financial support to several academic societies. We organise workshops and meetings designed to share the latest research, bridge fields and foster collaborations, and we help to build networks of researchers across the world through our online community sites: the Node, preLights and FocalPlane.

**Box 2. Useful links for our charitable and community activities**

Price transparency framework – https://journals.biologists.com/dmm/pages/transparent-metrics

Meeting Grants – https://www.biologists.com/grants/

Travelling Fellowships – https://www.biologists.com/travelling-fellowships/

DMM Conference Travel Grants – https://www.biologists.com/grants/dmm-conference-travel-grants/

Workshops and Journal Meetings – https://www.biologists.com/workshops/ and https://www.biologists.com/meetings/

the Node – https://thenode.biologists.com

preLights – https://prelights.biologists.com

FocalPlane – https://focalplane.biologists.com

## Supplementary Material

Supplementary information
