# Supplementary material for: Working to enhance the accessibility of Disease Models & Mechanisms
Source: Dis Model Mech. 2022 Jan 31;15(1):dmm049470. doi: 10.1242/dmm.049470 (PMC8822215; doi:10.1242/dmm.049470)
Supplement: Supplementary information [file dmm-15-049470-s1.pdf]

Reviewers for Disease Models & Mechanisms 2021

Omar Abdel-Wahab, Memorial Sloan Kettering Cancer Center, USA

Soman Abraham, Duke University, USA

Juan Abraldes, University of Alberta, Canada

Pankaj Agrawal, Boston Children's Hospital and Harvard Medical School, USA

Imran Ahmad, The Beatson Institute for Cancer Research, UK

Shreeram Akilesh, University of Washington, USA

Kathryn Albers, University of Pittsburgh, USA

W. Ted Allison, University of Alberta, Canada

James Amatruda, Children's Hospital Los Angeles and University of Southern California, USA

Robert Henry Anderson, University College London, UK

Sarah Annesley, La Trobe University, Australia

Yoshitsugu Aoki, National Center of Neurology and Psychiatry, Japan

Charlie Arber, University College London, UK

Nur Arfian, Universitas Gadjah Mada, Indonesia

Duchon Arnaud, CNRS, France

Timothy Arnett, University College London, UK

Ruben Artero, University of Valencia, Spain

Helen Arthur, Newcastle University, UK

Peter Arthur Farraj, University of Cambridge, UK

Maxim Artyomov, Washington University, St. Louis, USA

Kazuhide Asakawa, Tokyo Medical University, Japan

Jonathan Astin, University of Auckland, New Zealand

Sophie Astrof, Rutgers University, USA

John Auchampach, Medical College of Wisconsin, USA

Erika Bach, New York University School of Medicine, USA

Wolfgang Baehr, University of Utah, USA

Michel Bagnat, Duke University, USA

Yidong Bai, UT Health San Antonio, USA

Hua Bai, Iowa State University, USA

Andrei Bakin, Roswell Park Cancer Institute, USA

Jeroen Bakkers, Hubrecht institute, The Netherlands

Nektarios Barabutis, University of Louisiana at Monroe, USA

Barbara Bardoni, CNRS, France

Ariadna Bargiela, University of Valencia, Spain

Rosa Barrio, CIC bioGUNE, Spain

Valdir Barth, Harvard Medical School, USA

Peter Bass, Drexel University, USA

Gillian Beamer, Tufts University, USA

Thomas Becker, University of Dresden, Germany

Richard Behringer, MD Anderson Cancer Center, USA

Dimitris Beis, Biomedical Research Foundation Academy of Athens, Greece

Paola Bellosta, University of Trento, Italy

Brian Belyea, University of Virginia, USA

Ottavia Benedicenti, The University of New Mexico, USA

Jason Berman, Children's Hospital of Eastern Ontario Research Institute/University of Ottawa, Canada

David Bilder, University of California-Berkeley, USA

Colin Bingle, The University of Sheffield Medical School, UK

Katja Birker, Sanford Burnham Prebys Medical Discovery Institute, USA

Ivana Bjedov, University College London, UK

Eric Blalock, University of Kentucky, USA

Maria Blasi, Duke University School of Medicine, USA

Marc Blondel, Inserm/UBO, France

Karen Blyth, The Beatson Institute for Cancer Research, UK

Steven Boeynaems, Stanford University, USA

Benjamin Bolker, McMaster University, Canada

George Booz, University of Mississippi Medical Center, USA

Alexander Borowsky, UC Davis Cancer Center, USA

Gwenola Boulday, INSERM, France

Luke Boulter, University of Edinburgh, UK

Thomas Brand, Imperial College London, UK

Miriam Braunstein, University of North Carolina Chapel Hill, USA

Derek Brazil, Queen's University Belfast, Wellcome-Wolfson Building, UK

Jeroen Bremer, University Medical Center Groningen, The Netherlands

Volker Briken, University of Maryland, USA

Fiona Brown, Monash University, Australia

Rebecca Brown, NIH, USA

Valerie Brunton, University of Edinburgh, UK

Marcela Buchtova, Institute of Animal Physiology and Genetics, Czech Republic

Rebecca Burdine, Princeton University, USA

Alexa Burger, University of Colorado, USA

Robert Burgess, The Jackson Laboratory, USA

Emma Burkitt Wright, Manchester Centre for Genomic Medicine, UK

David Burns, University College Cork, Ireland

Jeffrey Bush, University of California, San Francisco, USA

Ross Cagan, Institute of Cancer Sciences - University of Glasgow, UK

Carlo D. Caiaffa, Florida International University, USA

Dana Cairns, Tufts University, USA

Kim Caldwell, The University of Alabama, USA

Katie Campbell, University College London, USA

Kirk Campbell, Icahn School of Medicine at Mount Sinai, USA

Gabriela Caraveo Piso, Northwestern University, USA

Thomas Carroll, University of Texas Southwestern Medical Center, USA

Sergio Casas Tinto, CSIC-Cajal Institute, Spain

Yang Chai, University of Southern California, USA

Joe Chambers, Manchester University, USA

Danny Chan, The University of Hong Kong, Hong Kong

Jia-Feng Chang, En Chu Kong Hospital, New Taipei City, Taiwan

Gavin Chapman, Victor Chang Cardiac Research Institute, Australia

Maggie Chasse, Harvard Medical School, USA

Yi-Wen Chen, Children's National Research Institute, Washington DC, USA

Weiqin Chen, Augusta University, USA

Yee Lian Chew, Flinders University, Australia

Hsueh-Cheng Chiang, National Cheng Kung University, Taiwan

Julie Christianson, University of Kansas Medical Center, USA

Steven Clapcote, University of Leeds, UK

Ben Clarke, Francis Crick Institute, UK

James Clement, Jawaharlal Nehru Centre for Advanced Scientific Research, India

David Clouthier, University of Colorado, Denver, USA

Mark Cookson, NIH, USA

Dezerae Cox, University of Cambridge, UK

Marija Cvetanovic, University of Minnesota, USA

Lawrence David, Duke University School of Medicine, USA

Clare Davies, University of Birmingham, UK

James Davis, University of Iowa, USA

Francesca De Santa, IBBC-CNR, Italy

Peter de Witte, KU Leuven, Belgium

Sarah Debebe, The Hospital for Sick Children, Toronto, Canada

Christos Delidakis, IMBB, FORTH, Greece

Jeroen den Hertog, Hubrecht Institute, The Netherlands

Alice Denton, Imperial College London, UK

Martin Denvir, University of Edinburgh, UK

Rajendar Deora, The Ohio State University, USA

Michael Devine, Francis Crick Institute, UK

Jean-Paul di Rago, CNRS-Bordeaux University, France

Paula Dietrich, The University of Tennessee, USA

Ann Mae DiLeonardi, US Army Research Laboratory, USA

Marc Dionne, Imperial College London, UK

Patrick Dischinger, Van Andel Institute Graduate School, USA

Maria Doitsidou, University of Edinburgh, UK

Pedro Domingos, Universidade Nova de Lisboa - ITQB, Portugal

Olivier Dorchies, University of Geneva, Switzerland

Anca Dorhoi, Friedrich-Loeffler-Institut, Germany

Lukas Dow, Weill Cornell Medicine, USA

James Dowling, The Hospital for Sick Children, Canada

Karen Downs, University of Wisconsin-Madison, USA

Timothy Doyle, Stanford University, USA

Ioannis Dragatsis, University of Tennessee, USA

Monica Driscoll, Rutgers, The State University of New Jersey, USA

Nelita Du Plessis, Stellenbosch University, South Africa

Sally Dunwoodie, Victor Chang Cardiac Research Institute, Australia

Sebastian Dworkin, La Trobe University, Australia

Claire Edwards, University of Oxford, UK

Marc Egerman, Icahn School of Medicine at Mount Sinai, USA

Rachel Eiges, Shaare Zedek Medical Center, Israel

Judith Eisen, University of Oregon, USA

Florent Elefteriou, Baylor College of Medicine, USA

Nicole Endlich, Institute of Anatomy and Cell Biology, Germany

Kevin Ess, Vanderbilt University, USA

Alessandra Eva, IRCCS Istituto Giannina Gaslini, Italy

Christopher Fang-Yen, University of Pennsylvania, USA

Colin Farquharson, Roslin Institute, UK

David Feliciano, Clemson University, USA

Feifei Feng, College of Public Health, China

Pedro Fernandez-Funez, University of Minnesota Medical School, USA

Erika Fernandez-Vizarra, University of Glasgow, UK

Olivier Feron, Université Catholique de Louvain, Belgium

Anthony Filiano, Duke University School of Medicine, USA

Richard Finnell, Baylor College of Medicine, USA

Silvia Finnemann, Fordham University, USA

Rita Fior, Champalimaud Foundation, Portugal

Bernie Fischer, Duke University, USA

Elizabeth Fisher, University College London, UK

Edward Fisher, NYU School of Medicine, USA

Cintia Folgueira, CNIC, Spain

Juan Fons, King's College London, UK

Marcia Fontes, Champalimaud Foundation, Portugal

James Foster, John Hopkins University, USA

Neil Freedman, Duke University Medical Center, USA

Tamio Furuse, RIKEN, Japan

Davide Gabellini, IRCCS Ospedale San Raffaele, Italy

Rene Galindo, UT Southwestern Medical Center, USA

Rajashekhar Gangaraju, University of Tennessee, USA

Antonio Garcia, Universidad Autónoma de Madrid, Spain

Ramon Garcia-Escudero, CIEMAT, Spain

Jose Garcia-Perez, The University of Edinburgh, UK

Moshe Gatt, Hadassah Hebrew University Medical Center, Israel

Alfred George, Northwestern University, USA

Brandon Gheller, Harvard Medical School, USA

Penney Gilbert, University of Toronto, Canada

Aaron Gitler, Stanford University School of Medicine, USA

Dominika Golubczyk, University of Warmia and Mazury, Poland

Richard Gomer, Texas A&M University, USA

Estela Gomez, Columbia University, USA

Raul Gomez, Instituto de Investigación Sanitaria, Spain

Diego Gómez-Nicola, University of Southampton, UK

Shubha Govind, The City College of New York, USA

Nina Graffmann, Institute for Stem Cell Research and Regenerative Medicine, Germany

Ryan Gray, University of Texas at Austin, Dell Medical School, USA

Timothy Greenamyre, University of Pittsburgh, USA

Stuart Greenhill, Aston University, UK

Alecia Gross, University of Alabama at Birmingham, USA

Joe Grove, University of Glasgow, UK

Guoqiang Gu, Vanderbilt University Medical Center, USA

Carmen Guerra, CNIO, Spain

Monica Guma, University of California, San Diego, USA

Vandana Gupta, Brigham and Women's Hospital, Harvard Medical School, USA

Maximiliano Gutierrez, The Francis Crick Institute, UK

Chady Hakim, University of Missouri, USA

Andrew Hall, University of Edinburgh, UK

Martina Hallegger, Francis Crick Institute and University College London, UK

Penelope Hallett, McLean Hospital and Harvard Medical School, USA

Noemie Hamilton, The University of Sheffield, UK

Milka Hammaren, EMBL, Germany

Chrissy Hammond, University of Bristol, UK

Renzhi Han, The Ohio State University College of Medicine, USA

Zhe Han, University of Maryland School of Medicine, USA

Anthony Hannan, Florey Institute of Neuroscience and Mental Health, University of Melbourne, Australia

Gregory Harms, University Medical Center Mainz, Germany

Stephen Harrap, University of Melbourne, Australia

Raymond Harris, Vanderbilt University, USA

Yasumasa Hashimoto, National Institute of Neuroscience, Japan

Courtney Hatton, The Jackson Laboratory, USA

Shannon Hawkins, Indiana University, USA

Tarik Haydar, Children's National Hospital, Washington DC, USA

Zhengxiang He, Icahn School of Medicine at Mount Sinai, USA

Ellen Heber-Katz, Lankenau Institute for Medical Research, USA

Miep Helfrich, University of Aberdeen, UK

Michael Henderson, Van Andel Research Institute, USA

Gretl Hendrickx, University of Antwerp, Belgium

Ahlke Heydemann, University of Illinois at Chicago, USA

Anthony Hickey, RTI International, USA

Philip Hieter, University of British Columbia, Canada

Paul Higgins, Albany Medical College, USA

Susumu Hirabayashi, MRC London Institute of Medical Sciences, Imperial College London, UK

Ping-Chih Ho, University of Lausanne, Switzerland

James Hodge, University of Bristol, UK

Diane Hoffman-Kim, Brown University, USA

JEFF Holly, University of Bristol, UK

Pamela Hoodless, BC Cancer Agency, Canada

Yariv Houvras, Weill Cornell Medical College, USA

Chih-Yu Hsieh, Taipei Medical University, Taiwan

Toby Hurd, The University of Edinburgh, UK

Mark Huycke, Oklahoma University Health Sciences Center, USA

Robert Hynds, University College London, UK

Igor Iatsenko, Max Planck Institute for Infection Biology, Germany

Akihiro Ikeda, University of Wisconsin-Madison, USA

Mathew Inkman, Washington University School of Medicine in St Louis, USA

Gareth Inman, CRUK Beatson Institute, UK

Clare Isacke, Breakthrough Breast Cancer Research Centre, UK

Sachiko Iseki, Tokyo Medical and Dental University, Japan

Anthony Isles, Cardiff University, UK

Junichi Iwata, The University of Texas Health Science Center at Houston, USA

Juan Carlos Izpisua Belmonte, Salk Institute for Biological Sciences, USA

Claudia Jakubzick, Dartmouth Geisel School of Medicine, USA

Nicholas Jarjour, University of Minnesota, USA

Daniel Jarocz, Stanford University, USA

Parmjit Jat, University College London, UK

Babak Javid, University of California San Francisco, USA

Jacek Jaworski, International Institute of Molecular and Cell Biology, Poland

Ashley Jean, University of Southern California, USA

James Jepson, University College London, UK

Wei Jiao, 904th hospital of PLA, China

Monica Justice, The Hospital for Sick Children, Canada

Daniel Kaganovich, Hebrew University of Jerusalem, Israel

Siddhesh Kamat, Indian Institute of Science Education and Research, Pune, India

Seong Kang, Emory University, USA

Gautam Kao, University of Gothenburg, Sweden

Ravi Karra, Duke University, USA

Kentaro Kato, Kyorin University School of Medicine, Japan

Maria-Cristina Keightley, La Trobe University, Australia

Amit Khairnar, NIPER, Ahmedabad, India

Kamal Khanna, NYU Langone Health, USA

Spiro Khoury, Université de Poitiers, France

Zoha Kibar, University of Montreal, Canada

Hoon Kim, Sunchon National University, Korea, Republic of

Kerri Kinghorn, University College London, UK

Kirill Kiselyov, University of Pittsburgh, USA

Susanne Klaus, German Institute of Human Nutrition, Germany

Pierre Klein, University College London/Francis Crick Institute, UK

Jenny Klein, Boston University, USA

Thomas Knudsen, Environmental Protection Agency, USA

Shivali Kohli, University of Exeter, UK

Maria Kontaridis, Masonic Medical Research Institute, USA

Uwe Kornak, Charité – Universitätsmedizin Berlin, Germany

Ron Korstanje, The Jackson Laboratory, USA

Kyoko Koshibu, Nestle, Switzerland

Peter Koulen, University of Missouri - Kansas City, USA

Laurent Kremer, IRIM CNRS, France

Doris Kretzschmar, Oregon Health and Science University, USA

Marie Lagouge, Sorbonne University, France

Timothy Lai, The Chinese University of Hong Kong, Hong Kong

Nicole Lake, Yale University, USA

Patricia Lalor, University of Birmingham, UK

Gyanu Lamichhane, Johns Hopkins University, USA

Karen Lange, University College Dublin, Ireland

David Langenau, Massachusetts General Hospital, USA

Lionel Larue, Institut Curie, France

Jean Latge, Institute Pasteur, France

Elisabeth Lawrence, University of Bristol, UK

Gwenael Layec, University of Massachusetts, USA

Robert Layfield, University of Nottingham Medical School, UK

Matthew Layne, Boston University School of Medicine, USA

Lu Le, UT Southwestern Medical Center, USA

Clementine Le Magnen, University Hospital Basel, Switzerland

Alan Yueh-Luen Lee, National Health Research Institutes, Taiwan

Jiae Lee, University of Washington, USA

Gabsang Lee, Johns Hopkins University, USA

Frances Lefcort, Montana State University, USA

Stylianos Lefkopoulos, The Max Planck Institute, Germany

Helmar Lehmann, Center for Molecular Medicine Cologne, Germany

Harry Leitch, MRC London Institute of Medical Sciences, UK

Rachel Lennon, University of Manchester, UK

Georges Levesque, Laval University, Canada

Daniel Levic, Duke University, USA

Jean-Pierre Levraud, Institute Pasteur, France

Andrew Levy, Technion Israel Institute of Technology, Israel

Patrick Lewis, Royal Veterinary College, UK

Yun Li, The University of Toronto, Canada

Leanne Li, Francis Crick Institute, UK

Daniel Liedtke, University of Wurzburg, Germany

Jennie Lin, Northwestern Medicine, USA

Chris Link, University of Colorado, USA

Chun-Feng Liu, Soochow University, China

Renjing Liu, Victor Chang Cardiac Research Institute, Australia

Junlai Liu, University of California San Diego, USA

Zhaoyang Liu, University of Texas, Austin, USA

Esther Liu, Northwestern University, USA

Cecilia Lo, University of Pittsburgh, USA

James Loan, University of Edinburgh, UK

Hanns Lochmuller, CHEO Research Institute, Canada

Jeremy Lotto, Terry Fox Laboratory, Canada

Ben Lovely, University of Louisville, USA

Jie Lu, Affiliated Hospital of Qingdao University, China

Wei-Yu Lu, University of Edinburgh, UK

Alejandro Lucía, Universidad Europea de Madrid, Spain

Georges Lutfalla, Université Montpellier 2, France

Donna MacCallum, University of Aberdeen, UK

Diana Machado, MD Anderson Cancer Center, USA

Fernando Macian, Albert Einstein College of Medicine, USA

Calum MacRae, Brigham and Women's Hospital, Harvard Medical School, USA

Pierre Magistretti, King Abdullah University of Science and Technology, Saudi Arabia

Fransiska Malfait, Ghent University, Belgium

Bilal Malik, University College London, UK

Silvia Mandillo, IBBC-CNR, Italy

Virginie Mansuy-Aubert, Loyola University, USA

Leilei Mao, Shandong First Medical University, China

Stefan Marciniak, University of Cambridge, UK

Elaine Mardis, Nationwide Children's Hospital, USA

Kate Maresh, University College London, UK

Kurt Marsden, North Carolina State University, USA

David Martinez, The University of North Carolina at Chapel Hill, USA

Luke McAlary, University of Wollongong, Australia

Kim McCall, Boston University, USA

Sarah McFarlane, University of Calgary, Canada

Sue McGlashan, The University of Auckland, New Zealand

Iain McNeish, Imperial College London, UK

Rosaria Meccariello, Parthenope University of Naples, Italy

Renaud Mevel, Universitätsspital Basel, Switzerland

Hemmo Meyer, University of Duisburg-Essen, Germany

Yuxuan Miao, University of Chicago, USA

Dimitra Micha, VU University Medical Centre, The Netherlands

Dustin Middleton, Duke University, USA

James Minchin, University of Edinburgh, UK

Jeff Miner, Washington University School of Medicine, USA

Rajesh Miranda, Texas A&M, USA

Jennyfer Mitchell, The Colorado Clinical and Translational Sciences Institute, USA

Sally Moody, George Washington University, USA

Torben Moos, Aalborg University, Denmark

Julie Moreau, Monash University, Australia

Jennifer Morton, The Beatson Institute, UK

Weipeng Mu, University of North Carolina at Chapel Hill, USA

Alex Muir, University of Chicago, USA

Manuel Muñoz, Universidad Pablo de Olavide, Spain

Daniel Murphy, University of Glasgow, UK

Kevin Myant, University of Edinburgh, UK

Ryan Myers, University of Minnesota Medical School, USA

Michio Nagata, University of Tsukuba, Japan

Aaron Nagiel, Children's Hospital Los Angeles, USA

Sundershana Nair,

Arafath Najumdeen, CRUK Beatson Institute, UK

Masanori Nakagawa, Kyoto Prefectural University of Medicine, Japan

Tsutomu Nakahara, Kitasato University School of Pharmaceutical Sciences, Japan

Masanori Nakayama, Max Planck Institute for Heart and Lung Research, Germany

Richard Naylor, Manchester University, UK

Lauryn New, University of Leeds, UK

Teresa Niccoli, University College London, UK

James Nichols, University of Colorado, USA

Marko Nikolic, University College London, UK

Emily Noel, University of Sheffield, UK

Osumi Noriko, Tohoku University School of Medicine, Japan

Emily Oates, University of New South Wales, Australia

Fumiaki Obata, RIKEN Biosystems Dynamics Research, Japan

Karen Ocorr, Sanford Burnham Prebys Medical Discovery Institute, USA

Oeystein R. Brekk, Harvard University, USA

S. Paul Oh, Barrow Neurological Institute, USA

Anil Ojha, Wadsworth Center, Albany, USA

Hidehiro Oku, Osaka Medical College, Japan

James Orengo, Baylor College of Medicine, USA

Ryan O'Shaughnessy, Queen Mary University of London, UK

Laurie Ozelius, Massachusetts General Hospital, USA

Mario Pagani, University of Buenos Aires, Argentina

Wilhelm Palm, DKFZ, Germany

Vijay Pancholi, The Ohio State University College of Medicine, USA

Pertti Panula, University of Helsinki, Finland

Mataleena Parikka, Tampere University, Finland

Alex Parker, Universite de Montreal, Canada

Barbara Parsons, U.S. Food and Drug Administration, USA

Basant Patel, Indian Institute of Technology Hyderabad, India

Carmen Pedraza, Universidad de Malaga, Spain

Simona Pedrotti, HSR Research - San Raffaele, Italy

Mercedes M. Perez-Jimenez, Universidad Pablo de Olavide, Spain

Norbert Perrimon, Harvard Medical School, USA

Randall Peterson, University of Utah, USA

Yvan Petit, Ecole de Technologie Supérieure, Universite du Quebec, Canada

Richard Piercy, The Royal Veterinary College, UK

Oksana Piven, National Academy of Sciences of Ukraine, Ukraine

Martin R. Pollak, Brigham and Women's Hospital, USA

Steve Pollard, University of Edinburgh, UK

Linda Popplewell, Royal Holloway, University of London, UK

David Porras, University of León, Spain

James Poulter, University of Leeds, UK

Andrew Preston, University of Bath, UK

Iryna Prots, Friedrich-Alexander-Universität Erlangen-Nürnberg, Germany

Sergey Prykhozhij, CHEO Research Institute & University of Ottawa, USA

Charlie Pyle, Duke University School of Medicine, USA

Susan Quaggin, Northwestern University, USA

Jianwen Que, Columbia University, USA

David Raible, University of Washington, USA

Dorota Raj, University of Gothenburg, Sweden

Tennore Ramesh, University of Sheffield, UK

Alan Ramsay, King's College London, UK

Antonia Ratti, Università degli Studi di Milano, Italy

Amir Rattner, Johns Hopkins Medical Institute, USA

Katherine Rauen, University of California Davis, USA

Martina Rauner, Technische Universität Dresden, Germany

Patricio Ray, University of Virginia, USA

Roger Reeves, Johns Hopkins University School of Medicine, USA

Mindong Ren, NYU Grossman School of Medicine, USA

Jian-gang Ren, Children's Hospital of Philadelphia, USA

Stephen Renshaw, University of Sheffield, UK

Johan Richter, Lund University, Sweden

Silke Rickert-Sperling, Charité Universitätsmedizin Berlin, Germany

Thomas Riemensperger, University of Cologne, Germany

Michael Ristow, ETH Zurich, Switzerland

Jennifer Ritchie, University of Surrey, UK

Karine Rizzoti, The Francis Crick Institute, UK

Ryan Roberts, Nationwide Children's Hospital, USA

John Robinson, University of Rhode Island, USA

Emily Rocha, University of Pittsburgh, USA

Adela Rodriguez-Romero, Universidad Nacional Autonoma de Mexico, Mexico

Maria Rohm, Helmholtz Zentrum München, Germany

Randall Roper, Indiana University Purdue University, USA

Richard Rosch, King's College London, UK

Mathias Rosenfeldt, University of Wurzburg, Germany

Hendrik Rosewich, Georg August University, Germany

Emily Rosowski, Clemson University, USA

Amy Rumora, Columbia University, USA

Hyung Don Ryoo, New York University School of Medicine, USA

Guadalupe Sabio, National Cardiovascular Research Center (CNIC), Spain

Takuya Sakaguchi, Cleveland Clinic, USA

Irene Salinas, University of New Mexico, USA

Eric Samarut, Universite de Montreal, Canada

Owen Sansom, The Beatson Institute for Cancer Research, UK

Ismael Santa-Maria, Columbia University, USA

Hiroki Sasaguri, RIKEN Center for Brain Science, Japan

Toshiro Sato, Keio University, Japan

Martin Scaal, University of Cologne Medical School, Germany

Annette Schenck, Radboud University Medical Center, The Netherlands

Lea Scherschinski, Barrow Neurological Institute, USA

Ruth Scherz-Shouval, Weizmann Institute of Science, Israel

Johannes Schlachetzki, University of California, San Diego, USA

Alan Schmalstig, University of North Carolina at Chapel Hill, USA

Miriam Schmidts, University of Freiburg Medical Center, Germany

Eric Schon, Columbia University, USA

Ben Schumann, The Francis Crick Institute, UK

Ian Scott, The Hospital for Sick Children, Canada

Ekihiro Seki, Cedars-Sinai Medical Center, USA

JoAnn Sekiguchi, University of Michigan, USA

Robert Semple, University of Edinburgh, UK

Andrea Serio, The Francis Crick Institute, UK

Claudio Sette, University Cattolica del Sacro Cuore, Italy

George Sharbeen, University of New South Wales, Australia

Renat Shaykhiev, Weil Cornell Medical College, USA

Tatyana Shelkovnikova, University of Sheffield, UK

Jennifer Shin, Korea Advanced Institute of Science and Technology, Republic of Korea

Dawn Siegel, Stanford University, USA

Dirk Sieger, University of Edinburgh, UK

Arndt Siekmann, Perelman School of Medicine, University of Pennsylvania, USA

Mirre Simons, The University of Sheffield, UK

Sarika Singh, CSIR-CDRI, Lucknow, India

Amit Singhal, Agency for Science, Technology and Research (A*STAR) Singapore, Singapore

John Sled, The Hospital for Sick Children, Canada

Aoife Slyne, University College Cork, Ireland

Clare Smith, Duke University School of Medicine, USA

Kelly D. Smith, University of Washington, USA

Andrei Smolnikov, University of New South Wales, Australia

Juhoon So, University of Pittsburgh, USA

Disease Models & Mechanisms • Supplementary information

Masahiro Sonoshita, Hokkaido University, Japan

Michelle Southard-Smith, Vanderbilt University Medical Center, USA

Guillermo Spitzmaul, INIBIBB, Argentina

Ashley St. John, Duke NUS Medical School, Singapore

Trace Stay, Stanford University, USA

Matthew Steensma, Van Andel Institute, USA

Torsten Stein, FU-Berlin, Germany

Polina Stepensky, Hadassah University Medical Centre, Israel

Mike Stern, Rice University, USA

Amber Stratman, Washington University School of Medicine in St. Louis, USA

Paulina Strzelecka, Charité Universitätsmedizin Berlin, Germany

Ruifang Sui, Peking Union Medical College Hospital, China

Kate Sutherland, Walter and Eliza Hall Institute, Australia

Ann Sutherland, University of Virginia, USA

Fran Sverdrup, Saint Louis University School of Medicine, USA

Amanda Swain, Institute of Cancer Research, UK

Delfien Syx, Ghent University, Belgium

Cliff Taggart, Queen’s University Belfast, Northern Ireland

Toki Takahashi, Tokyo Medical University, Japan

Kazuo Takayama, Kyoto University, Japan

Kiichiro Taniguchi, Kyoto University, Japan

Tanja Nielsen, Sanford Burnham Prebys Medical Discovery Institute, USA

Jason Tennessen, Indiana University, USA

Valeria Tiranti, Carlo Besta Neurological Institute, IRCCS, Italy

David Tobin, Duke University Medical Center, USA

Wei Tong, The Children’s Hospital of Philadelphia, USA

Alessio Torcinaro, IBBC-CNR, Italy

Anna Törnqvist, University of Gothenburg, Sweden

Reidun Torp, University of Oslo, Norway

Andrew Tosolini, University College London, UK

Howard Trachtman, NYU Grossman School of Medicine, USA

Jana Travnickova, University of Edinburgh, UK

Deborah Tribouillard-Tanvier, Institut de Recherche du CNRS, Paris, France

Eirini Trompouki, Max Planck Institute of Immunology and Epigenetics, Germany

Abigail Tucker, King’s College London, UK

Jouni Uitto, Sidney Kimmel Medical College at Thomas Jefferson University, USA

Ram Babu Undi, University of Oklahoma, USA

Martin Valdearcos, University of California San Francisco Diabetes Center, USA

Raphael Valdivia, Duke University School of Medicine, USA

Tom Van Agtmael, University of Glasgow, UK

Warren Van Loggerenberg, University of Toronto, Canada

Maaike van Putten, Leiden University Medical Center, The Netherlands

Boyd van Reijmersdal, Radboud University Medical Centre, The Netherlands

Roberto Vanacore, Vanderbilt University Medical Centre, USA

Greetje Vande Velde, KU Leuven, Belgium

Rajanikanth Vangipurapu, Saint Louis University School of Medicine, USA

Neeti Vashi, The Hospital for Sick Children, Canada

A. Catalina Vélez-Ortega, University of Kentucky, USA

Kartik Venkatachalam, The University of Texas Health Science Center at Houston, USA

Esther Verheyen, Simon Fraser University, Canada

Francesc Villarroya, Universitat de Barcelona, Spain

Petr Vodicka, Institute of Animal Physiology and Genetics, Czech Republic

Jakob Voelkl, JKU-Linz, Austria

Douglas Vollrath, Stanford University School of Medicine, USA

Yogesh Wairkar, University of Texas Medical Branch at Galveston, USA

Lucas Waltzer, GReD, CNRS, France

Hui-Li Wang, Hefei University of Technology, China

Brandon Warren, University of Florida, USA

John Warrick, University of Richmond, USA

Conrad Weihl, Barnes-Jewish Hospital, USA

Astrid Weins, Brigham and Women’s Hospital, USA

Dominic Wells, Royal Veterinary College, UK

David Westaway, University of Alberta, Canada

Ann Wheeler, University of Edinburgh, UK

Thomas Wilkie, UT Southwestern Medical Center at Dallas, USA

Matthew Winter, University of Exeter, UK

Marek Wolski, Medical University of Warsaw, Poland

Garry Wong, University of Macau, Macao

Zeng-Jin Yang, Johns Hopkins University, USA

Marielle Yohe, NCI, NIH, USA

Payal Yokota, New York University Langone Health, USA

Mark Yorek, University of Iowa, USA

M. James You, MD Anderson Cancer Center, USA

Sarah Zankar, The Ottawa Hospital, Canada

Sheng Zhang, University of Texas, USA

Jin Zhang, Washington University School of Medicine, USA

Haining Zhu, University of Kentucky, USA

Xianjun Zhu, University of Electronic Science and Technology of China, China

Oliver Ziff, The Francis Crick Institute, UK

Anna Zinovyeva, Kansas State University, USA

Leonard Zon, Harvard Stem Cell Institute, USA
